# Drug Sensitivity Testing in Osteosarcoma: A Case Report

**DOI:** 10.3390/curroncol32050271

**Published:** 2025-05-07

**Authors:** Ines Lohse, Giselle Dutcher, Hassan Al-Ali, Warren Alperstein, Donald W. Coulter, Matteo Trucco, Jonathan C. Trent, Claes Wahlestedt

**Affiliations:** 1 Center for Therapeutic Innovation, Miller School of Medicine, University of Miami, Miami, FL 33136, USA; 2 Department of Psychiatry and Behavioral Sciences, Miller School of Medicine, University of Miami, Miami, FL 33136, USA; 3 Molecular Therapeutics Shared Resource, Sylvester Comprehensive Cancer Center, University of Miami, Miami, FL 33136, USA; 4 Department of Orthopedic Surgery, University of Pittsburgh, Pittsburgh, PA 15213, USA; 5 Sylvester Comprehensive Cancer Center, University of Miami, Miami, FL 33136, USA; giselle.dutcher@gacancer.com (G.D.); halali@med.miami.edu (H.A.-A.); walperstein@miami.edu (W.A.); truccom@ccf.org (M.T.); jtrent@med.miami.edu (J.C.T.); 6 Miami Project to Cure Paralysis, Miller School of Medicine, University of Miami, Miami, FL 33136, USA; 7 Peggy and Harold Katz Family Drug Discovery Center, Miller School of Medicine, University of Miami, Miami, FL 33136, USA; 8 Department of Pediatrics, University of Nebraska Medical Center, Omaha, NE 68198, USA; dwcoulter@unmc.edu

**Keywords:** osteosarcoma, drug sensitivity testing, precision medicine

## Abstract

Precision medicine approaches using ex-vivo drug sensitivity testing (DST) have received attention in the cancer research community as a means to improve treatment stratification in populations where multiple treatment attempts are not feasible, or no standard-of-care treatment exists, such as ultra-rare cancers with a significant clinical need for effective treatment options, like osteosarcoma. DST has the potential to supplement existing patient stratification approaches by providing tumor-specific response data to aid in treatment selection at the time of treatment decision. We present the case of a pediatric osteosarcoma patient who was evaluated using DST at the time of standard-of-care treatment to evaluate treatment sensitivity. The DST screen indicated significant treatment sensitivity to anthracyclines and methotrexate, consistent with the first-line standard-of-care therapy (MAP). Clinical follow-up showed treatment sensitivity to standard-of-care MAP treatment and pathology results of 90% necrosis. The present case shows that DST screening is feasible from a technical standpoint, can be performed in a clinically relevant time frame that does not delay treatment start, and provides personalized drug sensitivity information on clinically available agents, and the DST results align with the clinical treatment response.

## 1. Introduction

Osteosarcoma is the most common primary bone tumor but represents only 3% of childhood cancers [1]. Combination therapy with chemotherapy and surgery is the current standard of care. Overall survival at 5 years has plateaued at 60–70% for newly diagnosed patients with localized disease and at 30% for patients with metastatic disease [1,2,3,4] Treatment stratification based on next-generation sequencing has failed to elucidate which sarcomas are likely to respond to chemotherapy or correctly predict which drugs will be effective in treating a sarcoma patient [5]. Drug sensitivity testing (DST) has the potential to provide personalized plans based solely on the response of the specific patient’s tumor cells to a library of anticancer agents. Using a patient’s tumor tissue for an ex-vivo drug screen can potentially identify favorable and unfavorable compounds for treatment of malignancies [6,7,8]. We present the case of a pediatric patient with osteosarcoma whose tumor was evaluated using DST screening concurrently with standard-of-care treatment. The DST screen was performed to evaluate the feasibility as part of clinical routine and provide evidence for the use of drug sensitivity information for the treatment stratification of osteosarcoma patients.

## 2. Materials and Methods

### Drug Sensitivity Testing

Consent for DST was obtained for the patient at the University of Miami (UM) under the IRB-approved Defining Platforms for Individualized Cancer Treatment (DePICT) trial (IRB 20150989). A portion of the tumor was viably frozen using freezing media and transferred to the DST laboratory. Then, 3 g of tumor tissue was processed for DST testing. The tumor piece was thawed and mechanically minced followed by enzymatic digestion to generate a cancer cell suspension with approximately 92% viability. The screen was performed as described previously [6,7,8,9]. Briefly, the cell suspension was seeded on assay plates and exposed to the library of 215 FDA-approved anticancer agents (Table 1). All compounds are evaluated in dose response, covering a 20,000-fold dose range. After 72 h of treatment, cell viability was evaluated, and dose–response curves were generated for each agent. These curves were analyzed using the drug sensitivity testing (DST) algorithm described in Swords et al. [8] to produce a modified drug sensitivity scoring (DSS_mod_) value for each agent. The same library of agents was applied to primary healthy pediatric osteoblasts and analyzed in the same way in order to establish tumor specificity and to assess normal tissue toxicity. The threshold of significant response (cancer cell killing) was defined as DSSmod value ≥5. Lastly, the selective drug sensitivity scoring (sDSS_mod_) value for each drug was calculated by the formula sDSS_mod_ = DSS_mod_ (patient cancer cells) − DSS_mod_ (normal osteoblasts). The sDSS_mod_ value thus incorporates the efficacy, potency, and therapeutic index for each agent into a numerical metric that can be used to rank the tumor-specific toxicity of different agents.

## 3. Results

### 3.1. Case Presentation

A 15-year-old male patient presented to medical attention in January 2019 with left knee pain progressing from a dull ache to pain with activity over the past year. Plain radiographs at the time of evaluation showed a left distal femur lesion with periosteal reaction, and an MRI revealed a partially calcified mass with dimensions of 11.1 cm × 4.1 cm × 3.1 cm arising from the bone. The patient underwent a biopsy of the left femoral lesion, resulting in a diagnosis of high-grade osteosarcoma. Staging workup also revealed a 0.9 cm × 0.7 cm lesion concerning for metastatic disease in the left upper lobe of the lung. The patient began standard-of-care therapy with MAP (methotrexate, adriamycin, cisplatin) chemotherapy per Children’s Oncology Groups (COG) protocol AOST0331 in February 2019. After two cycles of MAP, the patient underwent surgical resection in April 2019 with limb salvage surgery and mega-prosthesis placement. Pathology showed >90% tumor necrosis and negative margins. The patient received four cycles of adjuvant MAP to complete the planned standard therapy of six cycles given over 29 weeks. Video-assisted thoracoscopic surgery was performed in July 2019 to excise the pulmonary lesion that was confirmed to be osteosarcoma. Concurrently, a needle biopsy of a suspicious lesion in the L1 vertebral body was negative for disease. The patient remained on routine surveillance imaging; PET/CT 6 months after the completion of therapy showed possible progression in L1, and further work-up revealed disease, which was resected and treated with proton beam radiation targeting L1 (66 grey) and six cycles of ifosfamide and etoposide, finishing in January 2021. He started levantinib 10 mg PO Q Day in January 2021 [10] levantinib was discontinued in June 2021 after a canal-duodenal fistula was identified and repaired by surgery. He is currently in remission, with no evidence of disease on imaging completed in December 2025.

### 3.2. DST Results

**Figure 1:**
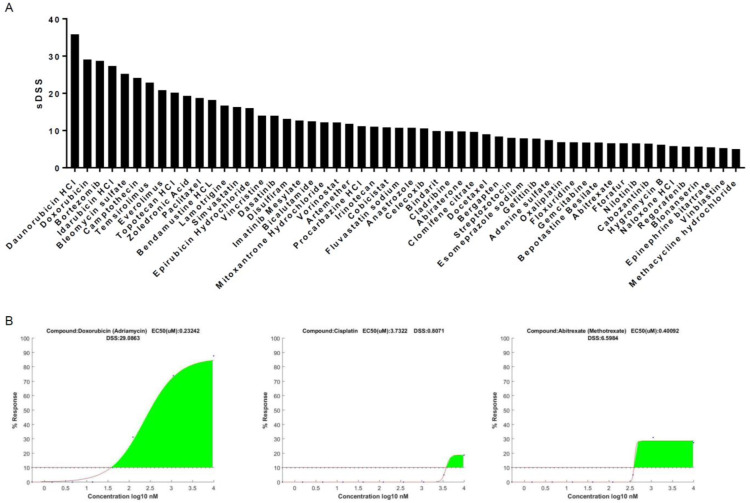
**Drug sensitivity testing results**. (**A**) Rank-ordered compounds displaying sDSSmod value above the threshold. (**B**) Dose–response curves of compounds used as part of MAP therapy.

**Figure 2:**
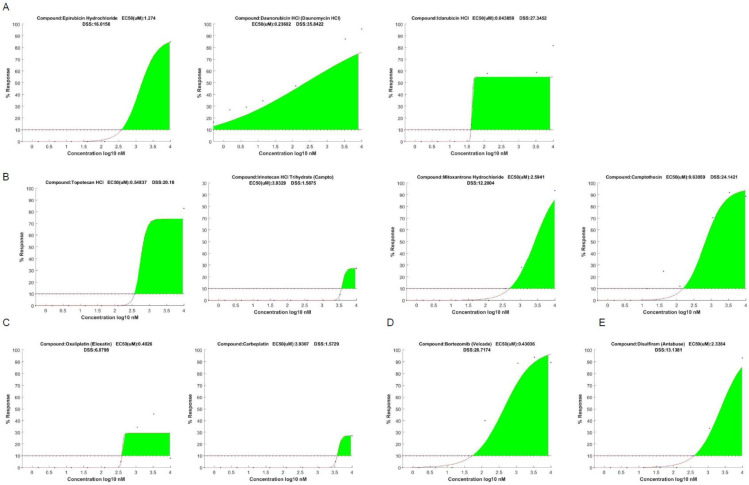
**Dose–response curves of compounds of interest**. Dose–response curves of (**A**) anthracyclines, (**B**) topoisomerase inhibitors, (**C**) platinum com-pounds, (**D**) bortezomib, and (**E**) disulfiram.

The tumor sample supplied sufficient material to support the full library screen and generate high-quality screening results. A total of 54 compounds displayed significant cancer cell killing above the threshold (Figure 1A, Table 2). Anthracyclines and topoisomerase inhibitors displayed high levels of treatment efficacy across the majority of compounds of their class present on the screen. All the anthracyclines represented in the screening library displayed significant treatment responses, namely, daunorubicin (DSS_mod_ = 35.84), doxorubicin (DSS_mod_ = 29.08), idarubicin (DSS_mod_ = 27.34), and epirubicin (DSS_mod_ = 16.01) (Figure 1A,B and Figure 2A, Table 2). Similarly, all of the topoisomerase inhibitors represented in the screening library displayed significant treatment responses, namely, camptothecin (DSS_mod_ = 24.14), irinotecan (DSS_mod_ = 11.04), mitoxantrone (DSS_mod_ = 12.20), and topotecan (DSS_mod_ = 20.18) (Figure 1A and Figure 2B, Table 2). In addition to anthracyclines and topoisomerase inhibitors, the antitumor antibiotics bleomycin (DSS_mod_ = 25.25) and zoledronic acid (DSS_mod_ = 19.31), everolimus (DSS_mod_ = 20.86), and temsirolimus (DSS_mod_ = 22.89) and the HDAC inhibitor Bortezomib (DSS_mod_ = 28.71) (Figure 2D) displayed significant treatment responses on the DST screen and were part of the top 10 compounds (Figure 2C, Table 2).

Other classical chemotherapy drugs, such as bendamustine, paclitaxel, vinblastine, vincristine, floxuridine, oxaliplatin, methotrexate, and gemcitabine, displayed DSS_mod_ values above the threshold; however, they were not among the top 10 compounds.

The screen also revealed significant treatment responses to disulfiram (DSS_mod_ = 13.13), a compound that has recently come into the focus of the osteosarcoma research community (Figure 2E) [9,10,11,12].

Of the compounds used clinically as part of the adjuvant MAP regimen, doxorubin displayed the largest treatment efficacy on the screen, with a DSS_mod_ value of 29.08 and around 85% maximum cell killing (Figure 1A,B). Both cisplatin and methotrexate (abitraxate) displayed only limited efficacy on the screen, with a DSS_mod_ of 3.73 and maximum cell killing below 20% and a DSS_mod_ of 6.59 and maximum cell killing around 30%, respectively (Figure 1A,B). The low response rate observed in response to treatment with cisplatin is consistent with the other platinum compounds present in the screening library, oxaliplatin and carboplatin. While oxaliplatin displays a response above the threshold, with a DSS_mod_ of 6.87 and maximum cell killing around 30%, carboplatin displays a response below the threshold, with a DSS_mod_ of 1.57 and maximum cell killing around 30%, (Figure 1A and Figure 2C).

**Figure 3:**
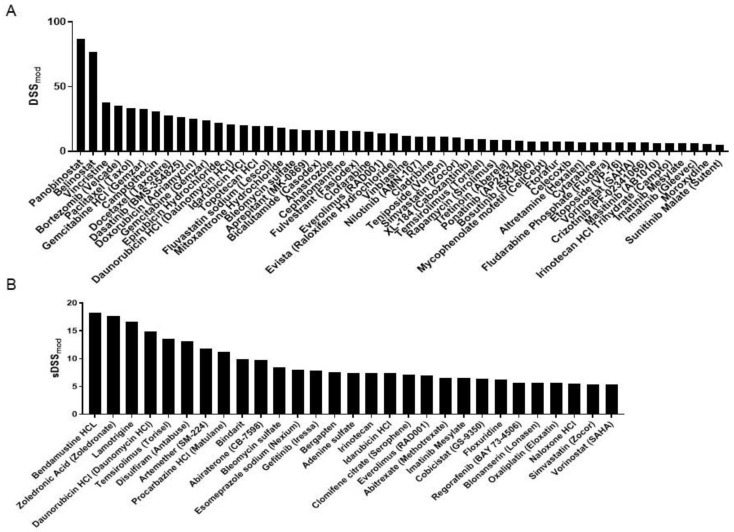
**Normal tissue toxicity**. Rank-ordered compounds displaying sDSSmod values above the threshold in (**A**) normal osteo-blasts and (**B**) patient sample.

Treatment-induced toxicities are of significant concern for pediatric cancer patients undergoing chemotherapy treatment, as they are often dose-limiting and can potentially affect patients for long periods of time. While exhaustive information exists on tissues where treatment-induced toxicities can be dose-limiting, such as blood and gut, little information exists for normal bone cells. To evaluate cancer specificity and normal tissue toxicity, the DST screen integrates normal osteoblast responses to treatment with the compound library, resulting in the sDSS_mod_ (Figure 3A). Positive values above the threshold indicate high levels of cancer specificity and low normal tissue toxicity. While the sDSS_mod_ should not be used to discount promising treatment candidates with high DSS_mod_ values, the sDSS_mod_ can distinguish between treatment with similar DSS_mod_ but vastly different toxicity profiles. This can be specifically important in patients where multiple continuous treatments are not possible. Significant treatment responses were observed in normal osteoblasts in response to treatment with the screening library (Figure 3A).

The analysis identified 29 such compounds (Figure 3B, Table 3). Integration of normal tissue toxicity significantly alters the sensitivity profile. This is not surprising considering the normal tissue toxicity clinically observed in response to classical chemotherapy as well as targeted agents. The sDSS_mod_ favors compounds such as antibiotics that generally display low toxicity. Notably, despite the high levels of toxicity generally associated with anthracyclines [12,13,14], daunorubicin (sDSS_mod_ = 14.92) and idarubicin (sDSS_mod_ = 7.33) show sDSS_mod_ values above the threshold. Doxorubicin (sDSS_mod_ = 3.84) on the other hand drops below the threshold due to its toxicity profile. The combination of low treatment efficacy and high levels of toxicity show cisplatin below the threshold. Although both methotrexate and oxaliplatin display sDSS_mod_ values above the threshold, both compounds are found at the lower end, with a sDSS_mod_ = 6.59 and sDSS_mod_ = 5.66, respectively. Surprisingly, disulfiram (sDSS_mod_ = 13.13) and temsirolimus (sDSS_mod_ = 13.58) display high levels of cancer specificity in this patient.

## 4. Discussion

Pediatric osteosarcoma remains a challenging disease. Despite numerous completed and ongoing clinical trials developed by national and international cooperative groups, survival rates have not changed significantly over the past 30 years [4,15,16,17]. The standard chemotherapy regimens for osteosarcoma are decades old and toxic, causing short- and long-term side effects in a majority of patients [18]. DST can provide clinicians with tumor-specific response data to aid in treatment selection. The feasibility and clinical utility of the DST platform have been previously demonstrated in a cohort of AML patients, showing that the ex-vivo drug sensitivity screen provides robust data allowing for rapid clinical decision-making [8]. The present study evaluates the technical feasibility of DST screening in a clinical sample of pediatric osteosarcoma. Additionally, we aim to provide evidence that this treatment stratification method can be performed in a clinically relevant time frame without delaying treatment start and generates patient-specific drug sensitivity profiles that align with clinical treatment responses. Although this case can add evidence supporting the further evaluation of the DST platform in larger osteosarcoma cohorts, this study is observational in nature, is limited to a single patient, and did not mandate DST-based treatment. Because this case was evaluated for feasibility rather than intent-to-treat, our results cannot evaluate whether a regimen outside of MAP therapy would have been more successful or whether a second screen of the relapse would display resistance to MAP therapy. This will be addressed in follow-up studies specifically addressing these points in larger patient cohorts.

The DST screen indicated significant treatment sensitivity to anthracyclines and methotrexate, consistent with the first-line standard-of-care therapy (MAP) and the pathology results of 90% necrosis. However, cisplatin, the third agent used in the MAP combination therapy, displayed low efficacy and DSSmod values below the threshold. A number of anticancer agents outside of the standard-of-care regimen displayed significant efficacy on the DST screen. The mTOR inhibitors everolimus and temsirolimus, as an example, displayed strong antitumor effects, consistent with predictions based on genomic studies and cell culture [19,20,21]. Of note, the cancer cells displayed significant treatment sensitivity to the drug disulfiram, a compound that has recently come into the focus of the osteosarcoma community. Although it is not commonly used for cancer treatment, disulfiram has shown anticancer effects in both in vitro and in vivo studies [9,22,23,24,25,26,27].

This pediatric osteosarcoma case shows that drug sensitivity testing of clinical osteosarcoma samples is feasible from a technical standpoint, can be performed in a clinically relevant time frame that does not delay treatment start, and provides personalized drug sensitivity information on clinically available agents, and the DST results align with the clinical treatment response. These data support the further investigation of the DST platform in larger clinical trials that will investigate safety and treatment efficacy in response to DST-guided therapy.

## Figures and Tables

**Table 1:** DST compound list. All anticancer agents included in the library are available through compassionate care and have shown anticancer effects in prior studies or are commonly used for supportive care.

Class	Compound
Alkylating agents	Bendamustine, Busulfan, Carboplatin, Cisplatin, Cyclophosphamide, Dacarbazine, lfosfamide, lomustine, Methazolastone, Oxaliplatin, Procarbazine, Streptozotocin
Antimetabolites	Azacitidine, Azaguanine-8, Capecitabine, Carmofur, Cladrabine, Clofarabine, Cytarabine, Decitabine, Febuxostat, Floxuridine, Fludarabine, Fluorouracil, Ftorafur, Gemcitabine, lonidamine, Mercaptopurine, Methotrexate, Nelarabine, Pemetrexed, Thioguanine
Antimitotics	10-Deacetylbaccatin, Cephalomannine, Docetaxel, Paclitaxel, Vinblastine, Vincristine
Antitumor antibiotics	Artemether, Azithromycin, Bacitracin, Bleomycin, Hygromycin B, Lincomycin, Methacycline, Ofloxacin
HDAC inhibitors	Belinostat, Panobinostat, Sodium Butyrate, Vorinostat
Hormone inhibitors	2-Methoxyestradiol, Abiraterone, Aminoglutethimide, Anastrozole, Bicalutamide, Clomifene Citrate, Diethylstilbestrol, Doxercalciferol, Enzalutamide, Exemestane, Flutamide, Fulvestrant, ltraconazole, Letrozole, Megestrol, Mifepristone, Paeoniflorin, Raloxifene, Tamoxifen, Toremifene, Triamcinolone
lmmunomodulators	Aspirin, Azathioprine, Bindarit, Cortisone, Celecoxib, Dexamethasone, Hydrocortisone, lmiquimod, Maraviroc, Meprednisone, Mizoribine, Mycophenolate, Phenylbutazone, Pimecrolimus, Pomalidomide, Prednisone, Sulindac, Tacrolimus, Thalidomide, Vinpocetine, Zileuton
Kinase inhibitors	Afatinib, Apatinib, Axitinib, Bosutinib, Cabozantinib, Crizotinib, Dasatinib, Erlotinib, lbrutinib, lmatinib, Lapatinib, Masitinib, Nilotinib, Pazopanib, Ponatinib, Regorafenib, Ruxolitinib, Sorafenib, Sunitinib, Tofacitinib, Vandetanib, Vemurafenib
Proteasome inhibitors	Bortezomib, Carfilzomib, Ubenimex
Rapalogs	Everolimus, Sirolimus, Temsirolimus
Topoisomerase 1/2 inhibitors	Camptothecin, Doxorubicin, Daunorubicin, Epirubicin, Etoposide, ldarubicin, lrinotecan, Mitoxantrone, Teniposide, Topotecan
Miscellaneous antineoplastics	Altretamine, Anagrelide, Bexarotene, Eltrombopag, Geniposide, Hydroxyurea, Mitotane, MLN4924, lsotretinoin, Tretinoin
Other	Adenine, Aprepitant, Atazanavir, Bepotastine, Bergapten, Blonanserin, Carbazochrome, Clorsulon, DAPT (GSI-IX), Disulfram, Dorzolamide, Ellagic acid, Epinephrine bitartrate, Esomeprazole, Ezetimibe, Flunarizine, Fluvastatin, Gadodiamide, Genistein, L-Arginine, Lamotrigine, Leucovorin, Linagliptin, Mesna, Mirabegron, Naloxone, Noscapine, Pamidronate, Pioglitazone, Ranolazine, Rosiglitazone, Orthovanadate, Temocapril, Tolbutamide, Valproic acid, Zoledronic acid, Vismodegib

**Table 2:** DSS_mod_ values.

Compound	DSS_mod_	Compound	DSS_mod_	Compound	DSS_mod_
Daunorubicin	35.84	Vorinostat	12.19	Bepotastine besilate	6.76
Doxorubicin	29.08	Artemether	11.82	Abitrexate	6.59
Bortezomib	28.71	Procarbazine	11.16	Ftorafur	6.58
Idarubicin	27.34	Irinotecan	11.04	Nilotinib	6.55
Bleomycin	25.25	Cobicistat	10.83	Cabozantinib	6.48
Camptothecin	24.14	Fluvastatin	10.74	Hygromycin b	6.17
Temsirolimus	22.89	Anastrozole	10.73	Naloxone hcl	5.80
Everolimus	20.86	Celecoxib	10.58	Regorafenib	5.67
Topotecan	20.18	Bindarit	9.88	Blonanserin	5.66
Zoledronic acid	19.31	Cladribine	9.83	Epinephrine bitartrate	5.50
Paclitaxel	18.75	Abiraterone	9.79	Vinblastine	5.27
Bendamustine	18.21	Clomifene citrate	9.63	Methacycline	5.02
Lamotrigine	16.70	Docetaxel	9.03		
Simvastatin	16.30	Bergapten	8.41		
Epirubicin	16.01	Streptozotocin	8.03		
Vincristine	14.01	Esomeprazole	7.92		
Dasatinib	13.98	Gefitinib	7.86		
Disulfiram	13.13	Adenine sulfate	7.45		
Imatinib mesylate	12.70	Oxaliplatin	6.87		
Bicalutamide	12.47	Floxuridine	6.85		
Mitoxantrone	12.20	Gemcitabine	6.76		

**Table 3:** sDSS_mod_ values.

Compound	sDSSmod	Compound	sDSSmod
Bendamustine HCL	18.21	Cobicistat	6.43
Zoledronic acid	17.66	Floxuridine	6.17
Lamotrigine	16.70	Regorafenib	5.67
Daunorubicin HCl	14.92	Blonanserin	5.66
Temsirolimus	13.58	Oxaliplatin	5.66
Disulfiram	13.13	Naloxone HCl	5.44
Artemether	11.82	Simvastatin	5.35
Procarbazine HCl	11.16	Vorinostat	5.35
Bindarit	9.88		
Abiraterone	9.79		
Bleomycin sulfate	8.49		
Esomeprazole sodium	7.92		
Gefitinib	7.86		
Bergapten	7.55		
Adenine sulfate	7.45		
Irinotecan	7.36		
Idarubicin HCl	7.33		
Clomifene citrate	7.08		
Everolimus	6.94		
Abitrexate	6.59		
Imatinib mesylate	6.48		

## Data Availability

The datasets used and/or analyzed during the current study are available from the corresponding author on reasonable request.
